# The top 100 manuscripts in emergency cardiac surgery. Potential role in cardiothoracic training. A bibliometric analysis

**DOI:** 10.1016/j.amsu.2019.05.002

**Published:** 2019-05-14

**Authors:** Rickesh B. Karsan, Arfon GMT. Powell, Prakash Nanjaiah, Dheeraj Mehta, Vasileious Valtzoglou

**Affiliations:** a Department of Cardiothoracic Surgery, University Hospital of Wales, Heath Park, Cardiff, CF14 4XW, UK; b Division of Cancer and Genetics, Cardiff University, Heath Park, Cardiff, CF14 4XW, UK; c Department of Surgery, University Hospital of Wales, Heath Park, Cardiff, CF14 4XW, UK

**Keywords:** Emergency cardiac surgery, Bibliometric analysis, Training, Citations

## Abstract

**Background:**

Emergency Cardiac Surgery (ECS) is a component of cardiothoracic training. Citations are considered to represent a papers influence. Bibliometric analyses allow us to identify the most influential work, and future research. We aim to highlight the key research themes within ECS and determine their potential impact on cardiothoracic training.

**Methods:**

Thomas Reuters Web of Science was searched using terms [Emergency AND Card* AND Surg*]. Results were ranked by citation and reviewed by a panel of cardiac surgeons to identify the top 100 cited papers relevant to ECS. Papers were analysed by topic, journal and impact. Regression analysis was used to determine a link between impact factor and scientific impact.

**Results:**

3823 papers were identified. Median citations for the top 100 was 88. The paper with the highest impact was by Nashef et al. focusing on the use of EuroSCORE (2043 citations). The Annals of Thoracic Surgery published most papers (n = 18:1778 citations). The European Journal of Cardiothoracic Surgery coveted the most citations (n = 2649). The USA published most papers (n = 55).The most ubiquitous topics were; risk stratification, circulatory support and aortic surgery. A positive relationship between journal impact fact and the scientific impact of manuscripts in ECS (P = 0.043) was deduced.

**Conclusion:**

This study is the first of its kind and identified the papers which are likely to the contribute most to training and understanding of ECS. A papers influence is partially determined by journal impact factor. Bibliometric analysis is a potent tool to identify surgical training needs.

## Highlights

• A Bibliometric analysis was performed, identifying influential publications in emergency cardiac surgery.
• Risk assessment, aortic surgery and circulatory support are key topics.
• Emergency surgery in pregnant patients was poorly represented.

## 1 Introduction

There are significant concerns in relation to emergency cardiac surgery (ECS), especially in respect to high morbidity and mortality within thoracic aneurysm repair and re-repair [1,2]. There is still uncertainty in such areas as to the best approaches to manage emergency cases and emergent research is essential to develop evidence based protocol to improve peri and post-operative outcomes.

Citation rankings highlight publications with the greatest influence [3]. Citation are gathered when publications are referenced by other peer-reviewed articles. It is clear to think that the more a piece of work is cited, the greater its impact in the scientific community. Bibliometric analysis or citation analysis are viewed as a marker of a papers influence. Such a process involves ranking an article or journal based on the number of received citations. In addition this tool is also used to rank journals based on their impact on the scientific community [3].

Many surgical specialities have utilised have used citation analysis to identify key research themes within the field including; general surgery [4] and orthopaedic surgery [5], as well as for surgical education [6]. Ellul and colleagues have previously used such an analysis to determine research themes that are most influential in understanding emergency abdominal surgery pathology and management to ultimately guide future citeable papers [7]. Within general cardiac surgery, it has been suggested that despite some flaws, bibliometric analysis has inherent merits to guiding future research [8].

This bibliometric analysis aimed to highlight key research themes within emergency cardiothoracic surgery that have had the greatest influence on developing management and understanding of related pathologies. Furthermore we hoped to demonstrate that the impact factor of a journal has a role in determining how often an article will be cited.

## 2 Methods

The Thomson Reuters Web of Science citation index database was searched using the terms [Emergency AND Cardiac AND Surg*]. The search was limited to English language, full manuscripts or abstracts. The results were ranked by citation number, using methods initially described by Paladugu and colleagues [4]. The final Web of Science search then was scrutinised by reviewing the identified abstracts, and articles found to have no relevance to ECS were excluded by 2 cardiac surgeons. The 100 most cited articles were identified and then evaluated by title, author and institution, department of the first author, topic, publication year and country.

A potential bias in this study type is that older articles will have more time to accrue citations. As a result a citation rate variable was created by dividing the number of citations by the number of years since publication, a method used by Ellul and colleagues [7]. The individual and 5 year impact factor of each journal were also recorded. Articles with the same number of citations were ranked based on the citation rate. Finally, regression analysis was performed to determine a potential relationship between, mean citations per journal and 5-year impact factor. Regression analysis was performed to evaluate the potential relationship between citation number and journal impact factor.

Exclusion criteria were articles in languages other than English and those unrelated to emergency cardiac surgery.

## 3 Results

The Web of Science database returned 3823 full English Language manuscripts. Table 1 provides a list of the top 100 papers ranked in order of citation [[9], [10], [11], [12], [13], [14], [15], [16], [17], [18], [19], [20], [21], [22], [23], [24], [25], [26], [27], [28], [29], [30], [31], [32], [33], [34], [35], [36], [37], [38], [39], [40], [41], [42], [43], [44], [45], [46], [47], [48], [49], [50], [51], [52], [53], [54], [55], [56], [57], [58], [59], [60], [61], [62], [63], [64], [65], [66], [67], [68], [69], [70], [71], [72], [73], [74], [75], [76], [77], [78], [79], [80], [81], [82], [83], [84], [85], [86], [87], [88], [89], [90], [91], [92], [93], [94], [95], [96], [97], [98], [99], [100], [101], [102], [103], [104], [105], [106], [107], [108]]. The median number of citations for the top 100 manuscripts was 88, with a mean of 135.16 (standard error 20.27); whilst the median yearly citation rate was 5.08, with a mean citation rate of 8.15 (standard error = 1.08). Of the top 100 manuscripts found, 39 had 100 citations or more as of 2018.

**Table 1:** The top 100 cited manuscripts in emergency cardiac surgery.

Rank	Citations	Average Citations per Year	First Author
1	2043	102.15	Nashef SAM [9]
2	1619	94.84	Hagan PG [10]
3	1223	48.92	Bickell WH [11]
4	630	23.33	Higgins TL [12]
5	391	20.58	Alexander KP [13]
6	362	30.17	Ferraris VA [14]
7	276	15.33	Edwards FH [15]
8	256	12.80	Chartier L [16]
9	225	20.45	Karkouti K [17]
10	211	15.07	Leacche M [18]
11	209	17.42	Jacobs AK [19]
12	188	9.89	Rhee PM [20]
13	171	8.55	Rozycki GS [21]
14	164	7.13	Rao V [22]
15	162	6.00	Plummer D [23]
16	159	6.63	Kimmel SE [24]
17	159	6.36	Ohman EM [25]
18	145	14.50	Wendt D [26]
19	140	5.00	Fulda G [27]
20	132	6.60	Ergin MA [28]
21	128	8.53	Hutfless R [29]
22	127	5.08	Borst HG [30]
23	125	10.42	Undre S [31]
24	123	6.15	Wong DT [32]
25	121	17.29	Shahian DM [33]
26	116	11.6	Haines NM [34]
27	114	6.33	Asensio JA [35]
28	113	4.91	Magovern JA [36]
29	111	7.93	Anguera I [37]
30	111	4.27	Dalton HJ [38]
31	110	4.07	Roudaut R [39]
32	106	5.05	Suen WS [40]
33	105	5.00	Asensio JA [41]
34	105	4.04	DeBono D [42]
35	104	5.47	Briguori C [43]
36	104	4.52	Parry AJ [44]
37	103	5.72	Roques F [45]
38	102	4.43	Magovern JA [46]
39	101	4.81	Li W [47]
40	99	4.30	Rozycki GS [48]
41	96	6.00	Tayal VS [49]
42	94	7.83	Megarbane B [50]
43	94	5.22	Avery GJ [51]
44	93	13.29	Smith PK [52]
45	93	4.23	Torchiana DF [53]
46	92	4.18	Gysi J [54]
47	91	4.33	Collier PE [55]
48	90	4.29	Trachiotis GD [56]
49	89	8.90	DiBardino DJ [57]
50	89	6.85	Hunt PA [58]
51	88	6.29	Collart F [59]
52	88	5.18	Schepens MA [60]
53	88	4.19	Gammie JS [61]
54	88	3.67	Moshkovitz Y [62]
55	87	5.44	Schwarz B [63]
56	87	4.58	Gruberg L [64]
57	87	4.58	Sprung J [65]
58	87	4.14	Asensio JA [66]
59	86	5.38	Charlesworth DC [67]
60	85	3.86	Duke T [68]
61	84	4.20	Magovern GJ [69]
62	83	4.37	Castillo JC [70]
63	81	10.13	Lange R [71]
64	81	10.13	White R [72]
65	79	3.16	Logeais Y [73]
66	76	10.86	Avalli L [74]
67	75	9.38	Gaca JG [75]
68	75	4.17	Neri E [76]
69	74	9.25	Harris KM [77]
70	74	4.93	Englberger L [78]
71	74	2.74	Sweeney MS [79]
72	72	4.24	Jamieson WRE [80]
73	70	8.75	Chikwe J [81]
74	69	6.90	Dunning J [82]
75	69	3.63	Suma H [83]
76	67	3.05	Munoz P [84]
77	66	5.08	Rastan AJ [85]
78	66	5.08	Degiannis E [86]
79	65	10.83	Lamhaut L [87]
80	65	5.00	Rastan AJ [88]
81	65	3.61	Bizzarri F [89]
82	64	3.05	Kontos MC [90]
83	63	4.85	Bossert T [91]
84	63	3.50	Smedira NG [92]
85	62	2.38	Dembitsky WP [93]
86	61	4.69	Schumacher H [94]
87	61	3.59	Hagl C [95]
88	61	2.65	Mavroudis C [96]
89	58	2.32	Kipfer B [97]
90	57	2.28	He GW [98]
91	56	7.00	Rylski B [99]
92	56	3.73	Manfredini R [100]
93	55	3.67	Patel NC [101]
94	55	3.44	Arnoni RT [102]
95	55	2.12	Buckman RF [103]
96	54	5.40	Zingone B [104]
97	54	2.35	Lin PJ [105]
98	53	5.30	Chandrasekhar S [106]
99	53	3.53	Tayal VS [107]
100	52	2.89	Yip HK [108]

The most cited article by Nashef et al. [9], reviewed surgical risk stratification via the EuroSCORE for patients undergoing cardiac surgery. This was published in the European Journal of Cardio-Thoracic Surgery in 1999 and has been cited 2043 times.

The oldest publication within the top 100, was published in 1991 by Fulda et al. investigating blunt traumatic rupture of the heart and pericardium and cited 140 times [27]. The most recent paper cited in this analysis was published in 2013 by Lamhaut et al., investigating the use of extracorporeal life support in the pre-hospital setting and was published in Resuscitation [87].

The 100 most cited papers were published by a total of 31 journals with the number of articles per journal ranging from 1 to 18 (Table 2). The Annals of Thoracic Surgery was found to have published the most articles (n = 18), whilst the European Journal of Cardiothoracic Surgery published 9 manuscripts but had the highest number of total citations (n = 2649). The journal with the highest impact factor was the New England Journal of Medicine (impact factor as of 2017 = 79.26; 5 year impact factor = 67.53) and published only 1 in the top 100, this however received 1223 citations. Chest was found to be the journal with the lowest impact factor was the Journal of Heart Valve Disease (impact factor as of 2017 = 0.715; 5 year impact factor = 0.883), and had published 1 article in the top 100.

**Table 2:** Journals with the top 100 cited Emergency Cardiac Surgery Manuscripts.

Journal title	Impact Factor as of 2017	5 Year Impact Factor	Number of Manuscripts in the Top 100	Total Number of citations	Mean number of citations
NEW ENGLAND JOURNAL OF MEDICINE	79.26	67.513	1	1223	1223
JAMA-JOURNAL OF THE AMERICAN MEDICAL ASSOCIATION	47.661	42.464	3	2408	802
EUROPEAN HEART JOURNAL	23.423	20.660	3	282	94
CIRCULATION	18.881	17.902	10	1403	140.3
JOURNAL OF THE AMERICAN COLLEGE OF CARDIOLOGY	16.834	18.737	5	990	198
INTENSIVE CARE MEDICINE	15.008	10.837	1	94	94
ANNALS OF SURGERY	9.203	9.097	1	99	99
CLINICAL INFECTIOUS DISEASES	9.117	8.970	1	67	67
CHEST	7.652	6.823	1	52	52
CRITICAL CARE MEDICINE	6.630	7.153	2	198	99
ANESTHESIOLOGY	6.523	6.546	2	210	105
RESUSCITATION	5.863	5.244	3	237	79
HEART	5.420	5.396	2	188	94
ANNALS OF EMERGENCY MEDICINE	5.008	5.441	2	226	113
JOURNAL OF THORACIC AND CARDIOVASCULAR SURGERY	4.880	4.334	13	1257	97
JOURNAL OF THE AMERICAN COLLEGE OF SURGEONS	4.767	4.972	3	389	129
ANNALS OF THORACIC SURGERY	3.780	3.854	18	1778	99
EUROPEAN JOURNAL OF CARDIO-THORACIC SURGERY	3.504	3.432	9	2649	294
ANAESTHESIA AND ANALGESIA	3.463	3.842	1	53	53
JOURNAL OF VASCULAR SURGERY	3.294	3.477	2	137	69
AMERICAN JOURNAL OF CARDIOLOGY	3.171	3.288	1	87	87
ANGIOLOGY	3.022	2.250	1	106	106
JOURNAL OF TRAUMA-INJURY INFECTION AND CRITICAL CARE	2.961	3.204	4	471	118
WORLD JOURNAL OF SURGERY	2.766	3.052	2	191	95.5
INJURY-INTERNATIONAL JOURNAL OF THE CARE OF THE INJURED	2.199	2.459	1	89	89
AMERICAN JOURNAL OF SURGERY	2.141	2.493	2	163	81.5
ASAIO JOURNAL	1.824	1.992	1	116	116
JOURNAL OF ULTRASOUND IN MEDICINE	1.530	1.889	1	53	53
JOURNAL OF CARDIOVASCULAR SURGERY	1.195	1.181	1	61	61
JOURNAL OF CARDIAC SURGERY	1.179	1.147	1	63	63
INTERNATIONAL JOURNAL OF ARTIFICIAL ORGANS	1.133	1.286	1	65	65
JOURNAL OF HEART VALVE DISEASE	0.715	0.883	1	103	103

**Fig. 1:**
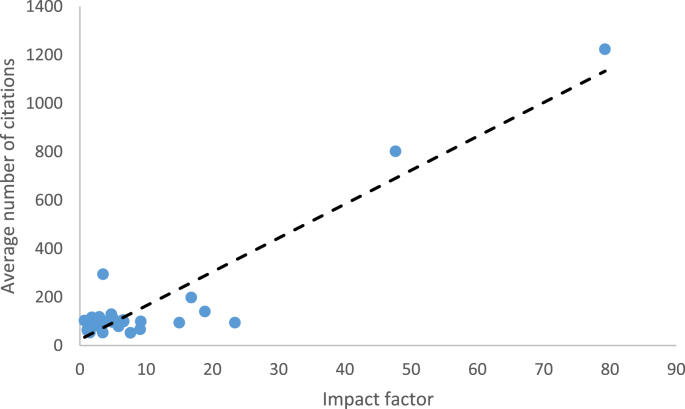
Relationship between impact factor and number of citations.

Fig. 1 highlights the potential relationship between a journals impact factor and citations received by an article. An overall positive association was deduced (r^2^ = 0.80, p = 0.043, CI 95% = 1.21–78.05) for the relationship between impact factor and citations received by and article.

The country with the highest number of publications in the top 100 manuscripts for ECS was the United States of American (n = 55), followed by Germany (n = 9). The United Kingdom contributed 5 manuscripts to the top 100 list. The countries with the lowest number of manuscripts in the top 100 were; the Netherlands, Austria, Australia, Israel, Japan, South Africa and Brazil (n = 1) (Table 3).

**Table 3:** Source countries of the top 100 articles in emergency cardiac surgery.

Country	Number of manuscripts in the top 100
USA	55
Germany	9
France	7
United Kingdom	5
Italy	5
Canada	4
China	3
Switzerland	3
Spain	2
Netherlands	1
Israel	1
Austria	1
Australia	1
Japan	1
South Africa	1
Brazil	1

The citation rate for the top 10 identified manuscripts related to ECS ranged from 102.5 for Nashef et al. (European system for cardiac operative risk evaluation (EuroSCORE)) [9] to 12.8 for Chartier et al. (Free-floating thrombi in the right heart - Diagnosis, management, and prognostic indexes in 38 consecutive patients) [16]. The United States of American had the most manuscripts in the top 100 (n = 7), the UK, France and Canada had 1 each.

**Fig. 2:**
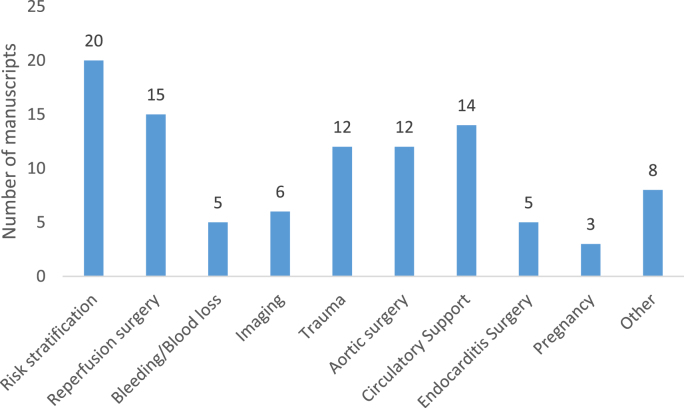
Number of manuscripts relating to individual topics with relevance to emergency cardiac surgery.

Fig. 2 highlights the number of manuscripts pertaining to a specific topic. Risk stratification was the most widely published topic with 20 manuscripts. This was followed by reperfusion surgery (n = 15). The use of circulatory support including ECMO and aortic surgery made up 12 manuscripts a piece within the top 100. Isolated manuscripts of other related topics looked into topics such as; the use of Cardiac Advanced Life Support (CALs) [82] and surgical management of massive pulmonary embolism [18].

## 4 Discussion

This bibliometric analysis is the first of its kind to identify the themes which have the greatest impact within the area of ECM. A myriad of pathologies, interventions and processes are encompassed by this diverse area of cardiac surgery. The most cited paper was by Nashef et al. [9] (2043 citations), published in the European Journal of Cardio-thoracic Surgery and focused on risk stratification and predicted mortality for patients undergoing major cardiac surgery. This focused on the use of a point scoring system to predict mortality and morbidity outcomes that can be used by clinicians. Risk stratification in cardiac surgery is the focus of other articles within the top 100 for ECS (n = 20). The second most cited paper by Hagan et al. [10], (1619 citations) published in Jama-Journal of the American Medical Association (impact factor 47.661) reported on the prevention, diagnosis and management of acute aortic dissection. The third most cited manuscript was by Bickell et al. [11] (1223) in the New England Journal of Medicine (impact factor 79.26) was a prospective trial comparing immediate and delayed fluid resuscitation in penetrating chest injuries, suggesting delayed fluid resuscitation prior to surgery improved outcomes.

There was substantial discrepancy in citation numbers for papers isolated in the top 100 for ECS. The top three papers, the citation numbers ranged from 2043 to 1223. The other thereafter ranged from 630 to 52. The median citation number was 88. This trend was not largely seen in other bibliometric analyses although Ellul et al. [7] showed a similar pattern when looking at emergency abdominal surgery. This substantiates suggestions that it reflects the relevance of the topic of risk stratification to non-emergency surgery disciplines, thus broadening the influence of the available literature. Furthermore, the citation numbers of the papers within the top 100 for ECS are significantly lower than for other surgical specialities. For example within orthopaedic surgery the three most cited articles identified by Kelly et al. had citation numbers of 1786,1146 and 1088 respectively, with a median citation number of 451 [109]. Similarly within cardiac surgery, O'Sullivan et al. illustrated a mean citation number of 457, with the top 4 most highly ranked manuscripts having citation numbers ranging from 1252 to 271 [8]. This looks to suggest relatively low research activity within the topic of ECS or a lack of available funding within this niche field, as well as significant challenges associated with conducting high quality trials in an emergency setting.

The topics covered within ECS demonstrate that a larger number relate to risk stratification (n = 20) of patients. This could likely represent the fact that within cardiac surgery as a whole, the risks of potential surgery needing cardio-pulmonary bypass are significant and with a move to less invasive strategies, a way to deduce management plans for higher risk patients is required so as to improve overall outcomes. The use of circulatory support, especially with the use of intra-aortic balloon pumps and extra-corporeal membrane oxygenation was also a significant topic of focus within the top 100 (n = 14). Emergency aortic surgery largely focused on surgical management of thoracic aortic dissection and chest trauma were also well covered within the top 100 (n = 12). Management of thoracic aortic dissections may have a larger focus due to the advent of endovascular repair and the potential for identifying key methods of management in term of open and endovascular repair.

Based on this analysis, it is evident that much of the research conducted was in the form of retrospective data search, cohort studies or case series. The use of randomised controlled trials is limited and may be as a result of formidable logistical challenges associated with the planning, and development of such clinical trials within the acute setting.

Influential manuscripts are more likely to have higher citation numbers, these citations form the basis of a journals impact factor. The journal impact factor in itself is a measure of the yearly average number of citations to recent articles published, and acts as a proxy of the importance of a journal within its field. Journals with a higher impact factor are thereby considered of being of a higher quality and more likely to publish the most influential manuscripts. The median impact factor for identified journals in this study was 3.78 with 64% of identified manuscripts published in journals with an impact factor of less than 5. Furthermore, the journals isolated with very high impact factor (79.26–15.008); New England Journal of Medicine, Jama-journal of The American Medical Association, European Heart journal, Circulation, Journal of the American College of Cardiology and Intensive Care Medicine represent on 23% of the top 100 publications.

Whilst the definition of impact factor gives an account to a journals specialist influence within the scientific community based on the citations its articles receive, the potential role of impact factor in relation to it facilitating higher citation rates for manuscripts and thus furthering a manuscripts scientific influence has not been established. Bibliometric studies by Paldugu et al. [4] and Ellul et al. [7] failed to show a relationship when considering how impact factor may positively or negatively influence the number of citations a manuscript gets. This study has highlighted that impact factor does indeed have a relationship with citation number (P = 0.043) in the realms of ECS (see Fig. 1). This study suggests that the greater a journals impact factor is, it the greater the number of average citations an article is likely to receive and thus a reflection of an articles greater influence within the scientific community. However it is interesting to note that journal impact factor was not always representative of total citation number at the level of individual papers for example, the highest ranked paper by Nashef et al. was published in the European Journal of Cardiothoracic Surgery (impact factor 3.504), which was the 18th ranked journal for impact factor in this study. Establishing the potential relationship between citation number and influence is a subject area that may require further work in the future, as a way to determine if such publications have in any way been integrated into modern ECS training within cardiothoracic surgery.

The findings of this study are potentially limited by a myriad of forms of bias. On such possibility is that articles may receive multiple citations as a result of self-citation, institutional or language bias. The high rate of publication within the USA has been mirrored in other studies included that looking at emergency abdominal surgery by Ellul and colleagues [7]. Other citation analyses studies by Powell et al. have also shown the same pattern [110,111]. Institutions within the USA may favourably cite local research, which may explain this pattern. Conversely, research culture within the US medical training encourages researchers to integrate research with their clinical practice. By limiting this research to English language articles, it is possible this effect was further exacerbated. Another point of possible bias is that older articles have greater time to accrue citations and thus not truly reflect research impact. To control for this, we calculated the citation rate per year (Table 1, Table 4). Even with this attempt at correction, lead-time for publications may result in more recent articles being under-represented in this study. A final point that may have contributed to bias within this study is that we limited the search to look only at first and senior author, and the institution of the first author. In many cases it is likely that there are several first authors who may have co-authored other papers in this top 100, as such they are likely under-represented in this current study format.

**Table 4:** Top 10 cited Emergency Cardiac Surgery manuscripts.

Rank	Citation Rate	First/Lead Author	Title	Institution	Country
1	102.15	Nashef SAM, EuroSCORE study group	European system for cardiac operative risk evaluation (EuroSCORE)	Royal Papworth Hospital, Cambridge	United Kingdom
2	94.84	Hagan PG	The International Registry of Acute Aortic Dissection (IRAD) - New insights into an old disease.	University of Michigan, Department of Cardiology	United States of America
3	48.92	Bickell WH	Immediate versus delayed fluid resuscitation for hypotensive patients with penetrating torso injuries	Baylor College of Medicine, Texas	United States of America
4	23.33	Higgins TL	Stratification of morbidity and mortality outcome by preoperative risk-factors in coronary-artery bypass patients – A clinical severity score	Cleveland Clinic Foundation, Ohio	United States of America
5	20.58	Alexander KP	Outcomes of cardiac surgery in patients age ≥ 80 years: Results from the National Cardiovascular Network	Duke Clinical Research Institute, North Carolina	United States of America
6	30.17	Ferraris VA, Society of Thoracic Surgeons Blood Conservation Guideline Task Force	Perioperative blood transfusion and blood conservation in cardiac surgery: The Society of Thoracic Surgeons and the Society of Cardiovascular Anesthesiologists Clinical Practice Guideline	University of Kentucky, Lexington	United states of America
7	15.33	Edwards FH	Prediction of operative mortality after valve replacement surgery	University of Florida, Division of Cardiothoracic Surgery	United States of America
8	12.8	Chartier L	Free-floating thrombi in the right heart - Diagnosis, management, and prognostic indexes in 38 consecutive patients	Hop Cardiologie, Serv Soins Intens Med & Reanimat Cardiaque, Lille	France
9	20.45	Karkouti K	Risk associated with preoperative anemia in cardiac surgery - A multicenter cohort study	Toronto General Hospital, Department of Anaesthesia	Canada
10	15.07	Leacche M	Modern surgical treatment of massive pulmonary embolism: Results in 47 consecutive patients	Brigham and Womans Hospital, Division of Cardiac Surgery, Boston	United States of America

## 5 Conclusion

The most highly cited papers in ECS cover a myriad of topics focusing largely on risk stratification, myocardial revascularisation, circulatory support and aortic aneurysm management. Emergency cardiac surgery in the pregnant patient and management of significant blood loss were poor reflected in this study and seem to relate to their clinical frequency of presentation and clinical burden. Despite the high-profile nature of ECS, this appears to be a relatively poorly researched area in cardiac surgery when reviewed from the angle of this bibliometric analysis, this is more than likely due to the difficulties in conducting high quality trials in such and acute setting. Nonetheless, ECS is a highly topical and pertinent subject within the realms of cardiac surgery and an essential part of training. This study has also highlighted that bibliometric analyses may be a quick and powerful tool to aide future cardiothoracic surgery training by ways of highlighting what the key subject areas within a sub-topic of a speciality are, and also what areas will require further research in the future. This bibliometric analysis provides insight into the most influential subjects and manuscripts in the speciality, and serve to show what subjects are topical, what areas need further research and ultimately what makes a citable and influential paper.

## Ethical approval

Not required.

## Sources of funding

Nil.

## Author contribution

**Rickesh B. Karsan**: Conceptualisation, methodology, formal analysis, investigation, writing - original draft, writing – review & editing, project administration. **Arfon G.M.T. Powell**: Methodology, formal analysis, resources, writing – review & editing, visualisation. **Prakash Nanjaiah**: Investigation, data curation. **Dheeraj Mehta**: Investigation, Supervision. **Vasileious Valtzoglou**: Investigation, data curation.

## Conflicts of interest

Nil.

## Reaserch registration number

Not required.

## Guarantor

Dr Rickesh B Karsan.

Department of Cardiothoracic Surgery, University Hospital of Wales, Heath Park Way, Cardiff, CF14 4XW.

Tel: 02920744620/+4407791804810, E-mail: rickesh.karsan@wales.nhs.uk/rk1727@my.bristol.ac.uk.

## Provenance and peer review

Not commissioned, externally peer reviewed.

## Abbreviations

ECS: Emergency cardiac surgery
UK: United Kingdom
USA: United States of America
